# THE ASSOCIATION OF PAIN WITH PAST AND FUTURE SELF-CONTINUITY

**DOI:** 10.1093/geroni/igz038.3186

**Published:** 2019-11-08

**Authors:** Gillian Fennell, Abby Pui Wang Yip, Cary Reid, Corinna Loeckenhoff

**Affiliations:** 1 University of Southern California, Los Angeles, California, United States; 2 Cornell University, Ithaca, New York, United States; 3 Weill Cornell Medicine, New York, New York, United States

## Abstract

Qualitative research on chronic pain patients’ subjective experiences has documented feelings of discontinuity between present and past selves due to changes in physical functioning and social roles. This investigation is the first to test the relationship between pain and self-continuity quantitatively and does so across two samples: Study 1 involved an adult community sample (n = 230, aged 18-87) and Study 2 involved a sample of older chronic pain patients (n = 145, aged 45-94). We explored potential differences for proximal versus distant selves and past versus future selves. In both studies, pain magnitude was negatively associated with average self-continuity (ps <.05), although the effect was selectively driven by future self-continuity in Study 1 (p < .01) and past self-continuity in Study 2 (p < .01). Additionally, in Study 2, recency of pain onset was negatively associated with past self-continuity (p < .001), but not with future self-continuity (p = .47). These findings suggest that chronic pain may be detrimental to self-continuity, with some variability linked to magnitude and chronicity of the pain. Health care providers may want to monitor their patients for feelings of disconnectedness with past and future selves. Future research is needed to identify therapeutic strategies that promote a continuous sense of self in spite of pain-related challenges.

